# Identification and validation of novel reference genes for bovine respiratory and lymphoid tissues using public transcriptomes and BRSV challenge model

**DOI:** 10.1371/journal.pone.0352137

**Published:** 2026-07-21

**Authors:** Cassandra D. Barber, Santiago Cornejo, Merrilee Thoresen, Matthew A. Scott, Amelia R. Woolums

**Affiliations:** 1 Mississippi State University, Mississippi, United States of America; 2 Veterinary Education, Research, and Outreach (VERO) Program, Texas A&M University, Canyon, Texas, United States of America; Cornell University, UNITED STATES OF AMERICA

## Abstract

RT-qPCR can be employed to quantify target gene expression normalized to reference genes that are expected to remain stable across experimental conditions. However, previously reported reference genes may not be stably expressed across all experimental conditions and individuals. This research identified novel candidate reference genes from publicly available healthy bovine tissue transcriptomes and subsequently developed and validated primers for five candidate reference genes. Forty-one transcriptomes were obtained from the NCBI Gene Expression Omnibus (GEO) from apparently healthy *Bos taurus* samples: bone marrow, bronchial lymph nodes (LN), mesenteric LN, kidney, liver, lung, nasal epithelium, unspecified LN, spleen, thymus, and trachea. Bioinformatic analysis of each dataset was performed using FastQC, Trimmomatic, STAR, and R, with library normalization carried out using the Relative Log Expression (RLE) method and normalized gene counts converted to log2-CPM values. The values for each gene across the transcriptomes were ranked by coefficient of variation (CV). The top 200 genes ranked by CV were analyzed using RefFinder for inter-tissue stability. Five candidates were selected: DDX23, NMT1, CRNKL1, TMEM183A, and UBE2Q1. To validate stability, RT-qPCR was used to assess the stability of these genes in tissues from seven bovine respiratory syncytial virus-infected calves and two healthy calves, including lung, bronchus, trachea, nasal epithelium, tracheobronchial LN, mediastinal LN, thymus, spleen, and bone marrow. The averaged CT values were analyzed with RefFinder. Comprehensive stability values identified CRNKL1 as the most or second most stable gene in 8/10 tissues, with variation in ranking of each reference gene across all tissues. This study identified a bioinformatic workflow and candidate reference genes, with primers validated for five genes which demonstrated stability across various bovine tissues.

## Introduction

Reverse transcriptase-quantitative polymerase chain reaction (RT-qPCR) is a powerful molecular biology technique used to quantify specific RNA in a sample with high sensitivity and specificity. In veterinary medicine, especially in large animal practice, RT-qPCR has become essential for infectious etiology testing. However, as scientific knowledge advances, there is increasing interest in biomarker or gene expression testing that detects host changes associated with disease states or outcomes. These gene expression studies indirectly assess translated proteins, since direct protein measurement is often costly [1]. Unlike classical PCR analyses, gene expression analysis that involves quantitative comparisons is more complex and requires proper methods and careful data analysis.

When performing a gene expression RT-qPCR experiment, selecting an appropriate data analysis method is imperative and is typically performed in one of two ways: absolute quantification or relative quantification. Absolute quantification requires a standard curve with known concentrations of target DNA. For target genes without commercially available standards, a plasmid containing the target gene can be constructed, but this can be an unnecessary extra step if the goal is to identify the overall trend in gene regulation (i.e., upregulated vs. downregulated). In this case, one can perform relative quantification normalizing to constitutively expressed genes, which allows the trend in expression of the target gene to be identified [2,3]. Historically, genes used for this normalization method were in the category of “housekeeping gene” and were defined as a gene required for cell maintenance [4]. However, new studies have shown that the expression of genes commonly targeted as endogenous controls (i.e., glyceraldehyde-3-phosphate dehydrogenase (GAPDH) and beta actin (ACTB)) may vary under different biological conditions [5,6]. Therefore, it is imperative to identify stable genes under each biological or experimental condition and to validate their stability to enable accurate and reproducible gene-expression analyses.

A common approach to identifying candidate reference genes in previous scientific literature, including bovine research, is to choose eight to ten candidate reference genes from existing studies [7–10]. While some researchers have successfully identified stable candidates, most conclude that the commonly used GAPDH and ACTB lack stability. Therefore, the question is whether there are novel gene candidates that exhibit greater stability and have not been previously recognized. Prior work in plant, microbial, and human cancer research has identified stably expressed genes in transcriptomic data generated by RNA sequencing (RNA-seq) [11–14]. RNA-seq experiments identify all RNA species in a sample and ultimately allow for the identification and relative quantification of genes/transcripts for detection of differential or stable expression [15]. Given the growing availability of publicly accessible transcriptomic datasets, we aimed to evaluate whether RNA-seq data can be used to identify a new candidate reference gene panel, which, to the authors’ knowledge, would be the first such effort in a livestock species.

Once gene candidates are identified, their stability can be assessed using stability calculations with programs such as NormFinder, GeNorm, BestKeeper, and DeltaCT [10,16–18]. All four calculations can be performed on the publicly available website RefFinder. Once stability calculations are completed, the stability values from each program are averaged to yield a comprehensive average stability value [19]. NormFinder identifies stability using a model-based approach that accounts for inter- and intra-group variability, yielding a stability value with the lowest value indicating the least variability and thus the most stable gene [16]. GeNorm and BestKeeper assess stability using a pairwise comparison between a gene of interest and all other control genes [10,17]. DeltaCT evaluates stability by determining the mean difference among reference genes; the most stable have the lowest mean standard deviation of CT changes [18]. Therefore, genes with values reflecting high stability can be used as reference genes to calculate fold changes for target genes in RT-qPCR experiments.

This study had two main objectives: first, to identify the most stable reference genes in healthy bovine tissues from publicly available transcriptomes, providing a useful resource for researchers conducting gene expression analyses across various studies. Ultimately, this aims to offer a starting point for those investigating bovine samples of respiratory and lymphoid origins, as well as liver and kidney tissues. Second, the study sought to explore the robustness of this reference gene selection strategy by investigating the stability of the candidate reference gene panel through RT-qPCR and stability analyses in respiratory and lymphoid tissues from calves challenged with the common viral respiratory pathogen, bovine respiratory syncytial virus (BRSV).

## Materials and methods

### Bioinformatic analysis identifying reference gene candidates

#### Obtaining publicly available transcriptomes.

**Fig 1:**
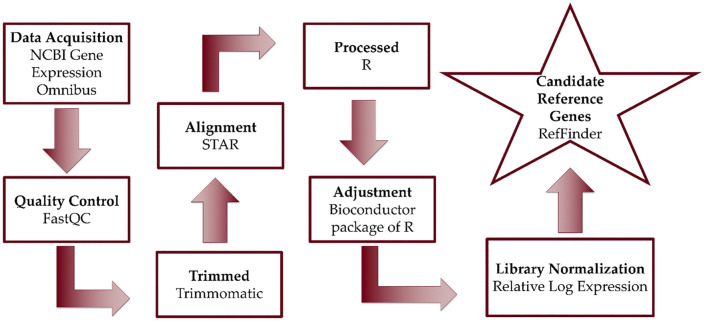
Bioinformatic workflow. Depiction of each step of the bioinformatic analysis used to identify candidate reference genes, to the point of identification of the relative expression (Log2) for each gene in each transcriptome. At that point the expression data for a subset of genes is evaluated with RefFinder to identify the stability value for each gene.

Utilizing NCBI Gene Expression Omnibus, 41 publicly available transcriptomes were identified from healthy bovine tissues (Table 1): bronchial lymph node (5), liver (11), kidney (6), lung (6), spleen (6), mesenteric lymph node (1), nasal mucosa (1), nasal pharynx (1), unspecified lymph node (1), bone marrow (1), trachea (1), and thymus (1) [20]. Bioinformatic workflow is depicted in Fig 1. It was assumed that the “bronchial” lymph nodes described in reference 22 indicates one of the tracheobronchial lymph nodes, which are located in close association with the trachea at the bifurcation of the mainstem bronchi, but this was not specified in that reference. The sample “lymph nodes” from reference 23 was not described more specifically in that reference and is thus described here simply as “unspecified lymph node”.

**Table 1:** Publicly available transcriptomes from clinically healthy *Bos Taurus* cattle.

Number of Individuals	Breed	Age	Sex	Tissues	Number of Transcriptomes	Number from NCBI Gene Omnibus	Citation
1	Holstein	2 years old	Female	Liver, Kidney, Lung, Mesenteric Lymph Node, Spleen	7	SRR1449243_44_45 SRR1449266_67_68 SRR1449269_71_72 SRR1449274_75_76 SRR1449287_88_89 SRR3081196 SRR3081200	[21]
3	Angus-Hereford	Unknown	Male	Bronchial Lymph Node	3	SRR2432553 SRR2432554 SRR2432555	[22]
1	Hereford	11 years old	Female	Nasal Mucosa, Unspecified lymph nodes, Lung, Bone Marrow, Trachea	5	SRR5363143 SRR5363146 SRR5363147 SRR5363153 SRR8703153	[23]
1	Holstein	3 years old	Male	Kidney, Liver, Lung	3	SRR10174881 SRR10174893 SRR10174899	[24]
1	Holstein	3 years old	Male	Kidney, Liver, Lung	3	SRR10174882 SRR10174894_95 SRR10174900	[24]
1	Holstein	3 years old	Female	Kidney, Liver, Lung	3	SRR10174883 SRR10174897 SRR10174901	[24]
1	Holstein	3 years old	Female	Kidney, Liver, Lung	3	SRR10174884_85 SRR10174898 SRR10174903	[24]
1	Hereford	180-day fetus	Male	Thymus	1	SRR042158	[25]
5	Holstein	1 year old	Un-known	Spleen, Liver	10	SRR8712456 SRR8712457 SRR8712458 SRR8712459 SRR8712460 SRR8712461 SRR8712462 SRR8712463 SRR8712464 SRR8712465	N/A
3	Holstein-Friesian	4 months old	Male	Bronchial Lymph Node	3	SRR9090335 SRR9090339 SRR9090340	[26]
Total: 16 Animals	4 Breeds	Fetus to Geriatric	Both Sexes		41		

#### Bioinformatic processing of transcriptomes.

To reduce bias from differences between single- and paired-end sequencing methods, each transcriptome was processed individually, and count tables were compiled. Raw sequenced read files for each dataset were quality assessed with FastQC v0.11.9 [27] and trimmed with Trimmomatic v0.39 [28]. Both single-end and paired-end reads were trimmed in a similar fashion, where read fragments were removed if leading and trailing base quality scores were less than 3. Reads were scanned with a 4-base pair sliding window and read segments below a minimum base quality score of 12 were removed, as were read fragments below a minimum length of 28 bases; residual adapters were also removed. Trimmed read fragments were mapped to the bovine reference assembly ARS-UCD2.0 via STAR v2.7.11a [29].

Briefly, reads were mapped and quantified through the guidance of Alternate Protocols 4 and 9 provided by Dobin and Gingeras, further generating raw gene-level count matrices for each transcriptome file via the “—quantMode GeneCounts” function embedded within STAR [30]. Raw gene counts generated for each transcriptome dataset were processed and analyzed in R v4.0.2 [31]. At this step, all transcriptomes were compiled into one data set in R. To adjust raw gene counts for uncontrolled variance, or batch effects, across each BioProject submission, the “ComBat_seq” empirical Bayesian function from the Bioconductor package sva v3.46.0 was utilized; this was applied to all sequencing libraries at the same time [32]. The eight batch factor IDs were used and assigned to each BioProject individually, except for the two BioProjects representing trachea and thymus samples. These two shared the same batch factor because they were the only BioProjects employing single-end pairing sequencing methods. Raw gene counts were then preprocessed and filtered using the filterByExpr function in the edgeR package as described by Chen et al., utilizing gene counts-per-million (CPM) of 1.0 across a minimum of 24 samples [33]. Library normalization was performed with the Relative Log Expression (RLE), using default settings [34]. Normalized gene counts were then log2-CPM converted, with the addition of a pseudocount of +1 to prevent log-transformation of any remaining zero counts. The absolute value of the coefficient of variance was then applied to each gene for every transcriptome and ranked in order of lowest coefficient of variance to highest.

#### Stability calculations via RefFinder.

Stability calculations were performed via a publicly available website from Xie et al. (2012) at https://www.ciidirsinaloa.com.mx/RefFinder-master/?type=reference, and it was determined that for the 41 transcriptomes, the program would evaluate up to 200 genes before reaching its computational capacity [19]. RefFinder calculates stability values from each of the previously published methods of DeltaCT, BestKeeper, NormFinder, and GeNorm, and then calculates the geometric mean of the stability values, called the comprehensive stability value [10,16–18]. The lower the stability values and, subsequently, the average stability value, the more stable the gene is; the top 22 most stable genes in this data set were retained for continued PCR analysis. Functions of these 22 genes are presented in S1 Table.

## Primer design

After identifying the stability rankings of the 22 most stably expressed genes identified via RefFinder, primers to CRNKL1, SPOP, NMT1, PIGU, YKT6, GZF1, MTERF4, CERS5, SNUPN, RANBP9, POP4, TMEM183A, DDX23, GNL2, FBXW11, and TTC4 were developed *in silico*. Previously published primers for UBE2Q1 were utilized [35] and all other primers were designed using Primer3 and BLAST software with the following criteria: 60 °C melting temperature, 40–60% GC content, 150–350 bp amplicon length, less than 3 guanidines on the 3 prime ends, primer oligonucleotide length of 18–25 bp, span exon-exon junctions, and less than 4 complementary of the 5’ prime to 3’ prime [36]; PCR conditions are described below.

### Exploration of stability of candidate reference genes

#### Stimulation of bovine neutrophils for positive control RNA.

To obtain positive control samples, eight apparently healthy, approximately 7-month-old *Bos taurus* Holstein bull calves were bled via the jugular vein into vacutainer tubes containing heparin, collecting a total of 300 mL. Blood was transferred in 10 mL aliquots to a 50 mL conical tube, diluted with 25 mL of Hank’s Buffered Balanced Salt Solution (HBSS, catalog #21–022-CV, Corning®, Manassas, VA), and 10 mL of density gradient medium (density = 1.077–1.080 g/mL, Lymphocyte Separation Medium, catalog #25–072-CV, Corning®, Manassas, VA) was added below the diluted whole blood. The mixture was centrifuged at 400 g for 30 minutes at room temperature without applying the brake, allowing cell separation. The plasma, peripheral blood mononuclear cells (PBMC), and density gradient layers were discarded, leaving the red blood cells (RBC) and granulocytes. The RBCs were lysed twice by adding 20 mL of sterile water (catalog #R500-01, Braun, Irvine, CA) for 5–10 seconds, followed by 2x Phosphate Buffered Saline (PBS, catalog #46–013-CM, Corning®, Manassas, VA), then mixed by inverting. To pellet the neutrophils, the samples were centrifuged at 200 g for 5 minutes. The neutrophils were resuspended in 1 mL of Dulbecco’s Modified Eagle’s Medium (DMEM, catalog #10–014-CM, Corning®, Manassas, VA) and pooled. Neutrophils were stimulated with lipopolysaccharide (LPS from Escherichia coli O55:B5, catalog #L4005, Sigma, Saint Louis, MO) by adding 10 µg per 1 mL of neutrophils and incubating at 37 °C for 30 minutes. The stimulated neutrophils were then frozen in cell pellets at −80 °C until RNA extraction, as described below.

This study’s animal handling, procedures, and care were reviewed and approved by the Mississippi State University Institutional Animal Care and Use Committee (IACUC) protocol 22–223.

#### Samples from healthy calves.

Two 2-month-old Holstein bull calves from a local dairy were assessed by physical exam and determined to be apparently healthy. On ultrasound, the lungs appeared free of abnormality with no consolidation observed. The next day, euthanasia of the calves was performed by intravenous barbiturate overdose (IACUC- approved protocol 21–073). With the assistance of a board-certified veterinary anatomic pathologist, necropsy of the calves was promptly performed, and tissue samples were snap-frozen in liquid nitrogen and stored at −80 °C until RNA extraction. Samples that were collected included nasal epithelium, trachea, bronchus, cranial and caudal lung, mediastinal lymph node (located in the mediastinum), tracheobronchial lymph node (located in close association with the trachea at the bifurcation of the mainstem bronchi), spleen, thymus, and bone marrow.

#### Samples from BRSV-infected calves.

Seven 2-month-old Holstein bull calves were acquired from a different dairy at birth and raised colostrum-deprived for an alternate research study (IACUC-approved protocol 19–368). These calves were raised until two months of age, at which time they were experimentally challenged by aerosol with 4 ml of bronchoalveolar lavage fluid previously harvested from a BRSV-challenged calf, which contained 4.4x10^3^ TCID_50_ units/ml of BRSV. On day 8 after challenge, all calves were euthanized via intravenous barbiturate overdose, and a gross necropsy was performed with the assistance of the same anatomic pathologist who supervised the necropsies of the healthy calves. Samples of nasal epithelium, trachea, bronchus, areas of normal and abnormal lung, mediastinal lymph node, tracheobronchial lymph node, spleen, thymus, and bone marrow were collected and snap-frozen in liquid nitrogen and stored at −80° C until RNA extraction. For infected calves, abnormal lung tissue was collected from the line of demarcation between normal and abnormal lung in the cranial lung, and normal tissue was collected from the caudodorsal lung. Samples were collected from healthy calves at the same sites for comparison.

#### RNA extraction and reverse transcription.

Frozen tissue samples were cryopulverized (BioPulverizer, catalog #59012N, BioSpec, Bartlesville, OK). RNA was extracted from the processed tissue samples and neutrophil cell pellets via a commercially available RNA extraction kit (RNeasy® Mini Kit, catalog #74104, QIAGEN, Valencia, CA) according to the manufacturer’s protocol, with homogenization (QIAshredder, catalog #79656, QIAGEN, Valencia, CA) and utilizing an on-column DNase treatment (RNase-Free DNase Set, catalog #79254, QIAGEN, Valencia, CA). The RNA was eluted in molecular-grade water (catalog #46–00-Cl, Corning, Manassas, VA) and kept on ice during quantity and quality assessments. The quantity of RNA was assessed using a benchtop fluorometer (Qubit 4 Fluorometer, catalog #Q33226, Invitrogen, Waltham, MA) with the Qubit RNA Broad Range Kit (catalog # Q10210, Invitrogen, Waltham, MA) and the quality was assessed using a microvolume spectrophotometer (Nanodrop 8000 Spectrophotometer, catalog #ND-8000-GL, Thermo Scientific, Waltham, MA) with goals of 260/280 ratios ~2.0 (S4 Table). Using a commercially available reverse transcription kit (qScript cDNA Supermix, catalog #95048−100, Quanta Biosciences, Beverly, MA) and using the manufacturer’s protocol, 1 μg of RNA was reverse transcribed into cDNA using a thermocycler (T100 Thermal Cycler, catalog #1861096, Bio-Rad Laboratories, Inc., Hercules, CA) and stored at −20°C until further analysis.

#### Conventional PCR for primer validation and RT-qPCR evaluation of candidate reference gene primers.

Conventional PCR for all prospective primers for each candidate reference gene target was performed using 10 ng of neutrophil cDNA (positive control), 10 ng of RNA (no reverse transcriptase control), or water (negative control or no template control). A commercially available classical PCR master mix (GoTaq® Green Master Mix, catalog #M7123, Promega, Madison, WI) was used according to the manufacturer’s protocol with the following conditions: incubation at 95 °C for 3 minutes, denature at 95 °C for 30 seconds, annealing at 60 °C or 64 °C for 1 minute, extension at 72 °C for 2 minutes, 30 cycles, and a final extension at 72 °C for 10 minutes. Primers described in the previous section were custom ordered from Sigma Aldrich (Darmstadt, Germany). The products were observed by 2.5% agarose (catalog #V3121, Promega, Madison, WI) gel electrophoresis with in-gel stain (SYBR™ Safe DNA Gel Stain, catalog #S33102, Invitrogen, Carlsbad CA) and imaged using an imaging system with UV transillumination and camera (ChemiDoc™ MP Imaging System, catalog #1708265, Bio-Rad Laboratories, Inc., Hercules, CA). The primers that did not have an amplicon in the no reverse transcriptase control and did yield a single positive band in the neutrophil cDNA sample were then selected for use in qPCR. Using SYBR green master mix (PerfCTa SYBR Green FastMix Low ROX, catalog #95074, Quanta Biosciences, Beverly, MA and Applied Biosystems 7500 Real-Time PCR machine, catalog #435986, Applied Biosystems™, Foster City, CA), the primers were manually tested with qPCR using 300 nM of each forward and reverse primer and 10ng of template (10 ul FastMix, 0.5 ul forward and reverse primers, 8 ul water, and 1ul cDNA). The qPCR conditions were 95 °C for 20 seconds, 40 cycles of 95 °C for 3 seconds, and 60 °C for 30 seconds. After the PCR reaction was complete, the melt curve was determined with treatment from 60 °C to 95 °C. For each gene target, positive control neutrophil cDNA, no reverse transcriptase control neutrophil RNA, and water were run first. If the primer had amplification in the cDNA sample only and a single peak on the melt curve, then that primer was assessed for efficiency. Lastly, efficiencies for target-specific primers were determined by using a range of starting concentrations of cDNA from 0.01 ng to 100 ng and calculated using the formula E = 10^(−1/slope)^ and percent efficiency = (E-1)*100%. Efficiency criteria were between 85–110%. RT-qPCR of all samples were performed in triplicate using 10 ng of cDNA per reaction.

#### Data analysis of RT-qPCR samples.

All RT-qPCR cDNA samples were run in technical triplicate. All technical replicates were assessed individually, and individual values were excluded if aberrant amplification curves were yielded or the C_t_ value was inconsistent with the remaining replicates. For any remaining samples with a standard deviation greater than 0.5, PCR was repeated. Abundances of the target genes were identified in Excel by calculating 2^-geometric mean of C_t_ values of all technical replicates for all samples. Where averages are described, the geometric mean was calculated. The raw C_t_ values were also assessed using RefFinder to identify the stability of each candidate reference gene from each tissue sample. GraphPad Prism v10.6.1 was used to generate the figures except for the principal component plot, for which R v4.5.1 and RStudio v2025.09.0 + 387 was used.

## Results

### Transcriptome analysis

**Fig 2:**
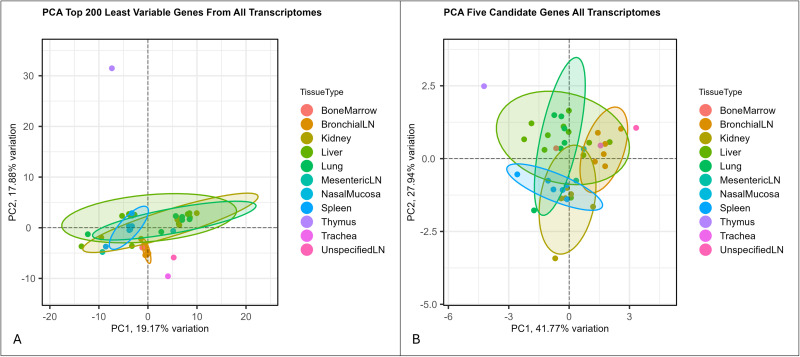
Principal Component Analyses. Principal component analyses using the Log2 CPM from transcriptomic data sets of the top 200 least variably expressed genes based on coefficient of variance (2A) and the five genes identified after PCR analysis was performed (2B). Ellipses are set at 80% confidence.

The transcriptome identification from NCBI Gene Expression Omnibus identified 41 transcriptomes of healthy cattle, including some that came from animals that served as untreated controls in experiments. As described in Table 1, transcriptomes were obtained from 16 animals across 4 cattle breeds, ranging from fetuses to geriatric cattle, both male and female. Due to the lack of more publicly available healthy bovine transcriptomes there was an unequal distribution of available tissue transcriptomes, including a total of bronchial lymph node (5), liver (11), kidney (6), lung (6), spleen (6), mesenteric lymph node (1), nasal mucosa (1), nasal pharynx (1), unspecified lymph node (1), bone marrow (1), trachea (1), and thymus (1). S2 Table contains the raw CPM counts of 13,842 genes ranked by lowest coefficient of variance per gene to highest and the stability values calculated from the 200 least variable genes via coefficient of variance calculation from RefFinder assessment. Stability values calculated by each program, and the comprehensive stability value, for the 200 most stably expressed genes are reported in S3 Table. Principal component analysis was performed to show that these tissues are not dissimilar to one another by overlapping and non-independent clustering with Fig 2A showing the 200 genes with the lowest variability and Fig 2B showing the 5 selected candidate reference genes. The resulting PCA plots show that most tissues overlap and do not cluster independently. Some datasets consisted of single samples, and ellipses could not be graphed for mesenteric lymph node, nasal mucosa, unspecified lymph node, bone marrow, trachea, and thymus. Technical variation in the trachea and thymus samples in Fig 2A could have led to their dissimilarity along the PC1 axis compared to the other data sets. Fig 2B is a PCA plot restricted to genes for which primers were developed, and with the smaller data set, fewer variables are calculated, allowing greater variance to be explained by PC1 (41.77%) and PC2 (27.94%). Consistent with Fig 2A, these samples are also similar to one another, as all ellipses overlap and do not show independent clustering. By refining the data input for these selected genes, the thymus and unspecified lymph node samples are closer to the ellipses, and the trachea is contained within the ellipses, indicating little to no dissimilarity of these tissues from the other tissues in terms of gene expression variance (Fig 2B). These PCA plots demonstrate that, regardless of tissue type, the identified transcriptomic gene expression remains consistent across tissues and samples, ultimately proposing stability among tissues of the candidate reference genes.

**Fig 3:**
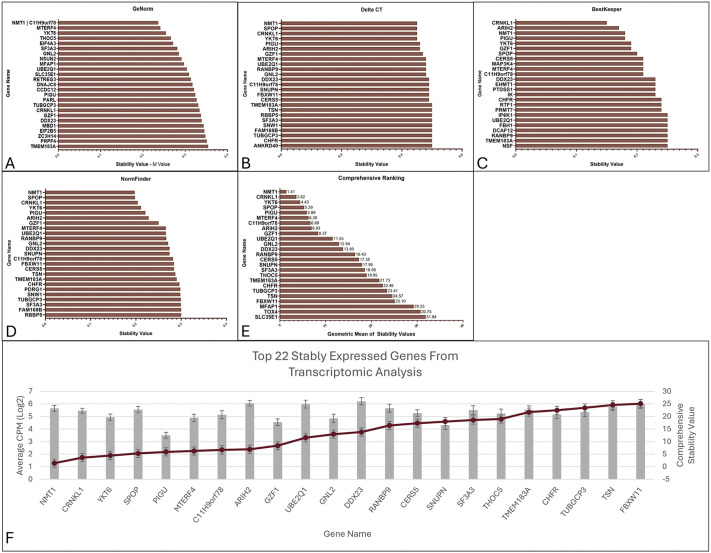
Top 22 most stably expressed genes by method, comprehensive ranking, and abundance. Stability values for each gene as calculated by the GeNorm method (3A); the NormFinder method (3B); the DeltaCT method (3C); and the BestKeeper method (3D). In these graphs, the most stable gene appears at the top, while the least stable is at the bottom. A lower stability value indicates greater stability. Fig 3E shows a comprehensive ranking of each gene calculated by the geometric mean of the stability values determined by each program. Fig 3F illustrates a graph of the top 22 most stable genes across 12 tissue types within 41 transcriptomes from apparently healthy cattle. The line and the right vertical axis depict the stability value for each gene, with the most stable on the left and the less stable on the right. The bars and the left axis represent gene abundance (copies per million) on a log2, plus the standard deviation.

After analyzing the top 200 genes with RefFinder, the genes with a comprehensive stability value of 25 or less were plotted in Figs 3A-D. Fig 3A-D demonstrates the stability values determined by each of the programs RefFinder uses to calculate the average stability value: GeNorm (3A), BestKeeper (3B), Delta CT (3C), and BestKeeper (3D). The average stability value calculated by RefFinder is shown in Fig 3E. Fig 3F demonstrates these 22 genes within the 41 transcriptomes ranked from lowest stability (left) to highest (right). Also graphed in Fig 3F is the average abundance (average Log2 copies per million + /- the standard deviation) of these genes within the samples. RefFinder identified the genes in order of lowest comprehensive stability value (most stable) to the highest comprehensive stability value (least stable): NMT1 < CRNKL1 < YKT6 < SPOP < PIGU < MTERF4 < C11H9orf78 < ARIH2 < GZF1 < UBE2Q1 < GNL2 < DDX23 < RANBP9 < CERS5 < SNUPN < SF3A3 < THOC5 < TMEM183A < CHFR < TUBGCP3 < TSN < FBXW11. These genes and their functions are described in S1 Table. The commonly used reference genes GAPDH and ACTB were not identified within the top 2000 genes ranked on the coefficient of variance and were ranked 5218 and 5002, respectively.

### PCR primer development

Primer design for most genes in the top 22 reference gene candidates was attempted. Our lab previously designed and validated a primer set for UBE2Q1, which was employed in this study [35]. Primers which met the design criteria could not be developed for PIGU, GZF1, SNUPN, RANBP9, and FBXW11 because they either failed to span exon-exon junctions, were prone to self-complementarity, or were likely to form primer dimers. As a result, they were excluded from the rest of the study. Primers that met the design requirements included CRNKL1, SPOP, NMT1, YKT6, MTERF4, CERS5, TMEM183A, DDX23, UBE2Q1, TTC4, and GNL2. Multiple primers were tested for SPOP (2), YKT6 (2), MTERF4 (2), CERS5 (3), TTC4 (1), and GNL2 (2), but suitable primers could not be developed. Details regarding the problems encountered during efforts to develop primers for these genes are described in S1 File. Therefore, the final primers identified were for CRNKL1, NMT1, TMEM183A, DDX23, and UBE2Q1, which met the primer design criteria, had optimized efficiency between 85–105%, and were specific to binding mRNA from the sample without binding genomic DNA. Novel reference gene candidate primer sets that were validated further are described in Table 2.

**Table 2:** Information regarding primer sets.

Gene Name	Forward Primer	Reverse Primer	Amplicon Size	GC content F/R	Efficiency (%)
CRNKL1	CAATCCTTGGCGATATTGAGCG	GCCTCTCTTCCTTTTCCTCACA	303	50/50	101.5
NMT1	GGTACTGGCATCGGTCCCTA	TCACCTCCCCGTTTGCATTC	300	60/55	104.3
TMEM183A	GAGCAAGTGTATGGGAGGATTGC	ACTGAGGATGCCACCAGTCAAA	305	52.1/50	97.4
DDX23	TCGGCATTGGTCCCAGAAAA	GATAGGCGTTGGCTCCTTGT	190	50/55	88.5
UBE2Q1	GCAACATCACGGAGTCAT	CAGCAGCCAAGTTAGGGT	75	55/57	101.8

**Fig 4:**
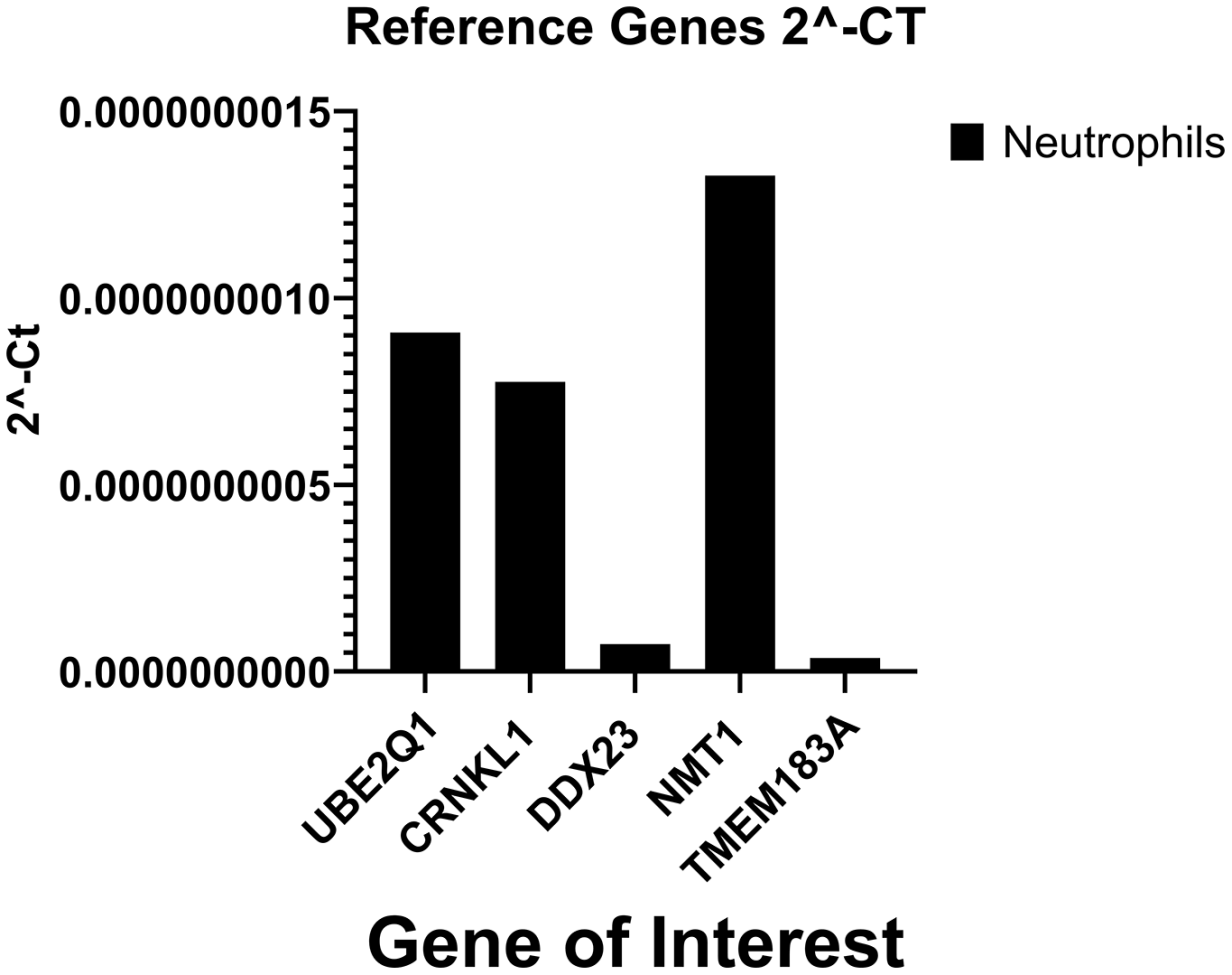
Reference gene expression by LPS-stimulated neutrophils. Fig 4 shows the expression abundance of each candidate reference gene in LPS-stimulated neutrophils. Samples were tested in triplicate, and the geometric mean was used to calculate abundance equaling 2^-averageCT.

Testing of the candidate reference gene primers in RT-qPCR of RNA from stimulated bovine neutrophils confirmed that the primers bound specifically to intended targets, as no product was amplified in the no reverse transcriptase control, and one pointed peak was identified on each of the melting curve plots, as shown in S1A-E Fig. Efficiencies for each primer set were obtained using five 10-fold dilutions of cDNA from the positive control sample of stimulated neutrophils. Table 2 describes the efficiencies for each primer set, which were all adequate, between 88.5–104.3%. The abundance (2^-C_t_) of expression of these genes in LPS-stimulated neutrophils is shown in Fig 4, with NMT1 having the highest abundance, followed by UBE2Q1, CRNKL1, DDX23, and TMEM183A.

### Exploration of stability of novel reference gene candidate primers within viral infected and non-infected calves

**Fig 5:**
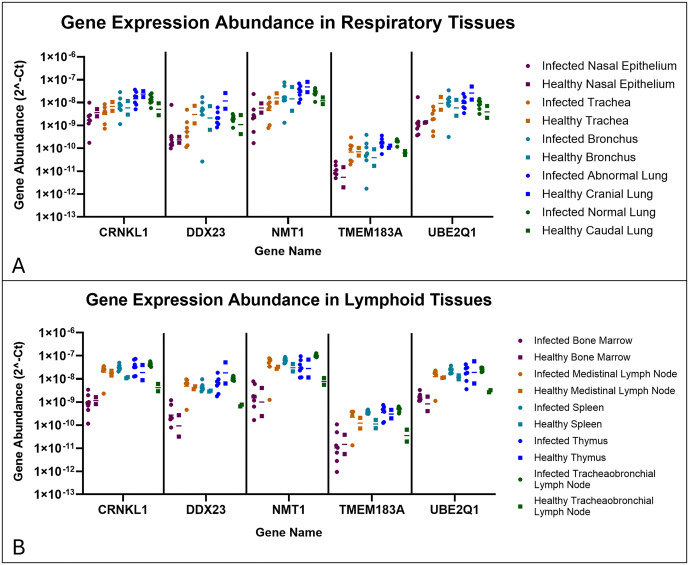
Gene expression abundance in healthy and BRSV-infected respiratory and lymphoid tissue as determined by RT-qPCR. Fig 5 shows RT-qPCR C_t_ values calculated as 2^-averageCT. Tissues were collected at necropsy from healthy (n = 2) and BRSV-challenged (n = 7) calves. RT-qPCR was performed in triplicate for all samples, with the average for each biological replicate shown as points on the scatter plot, and the horizontal line among each set of points indicating the mean of the respective data set. Fig 5A demonstrates the results for respiratory tissues, and  Fig 5B demonstrates the results for lymphoid tissues.

**Fig 6:**
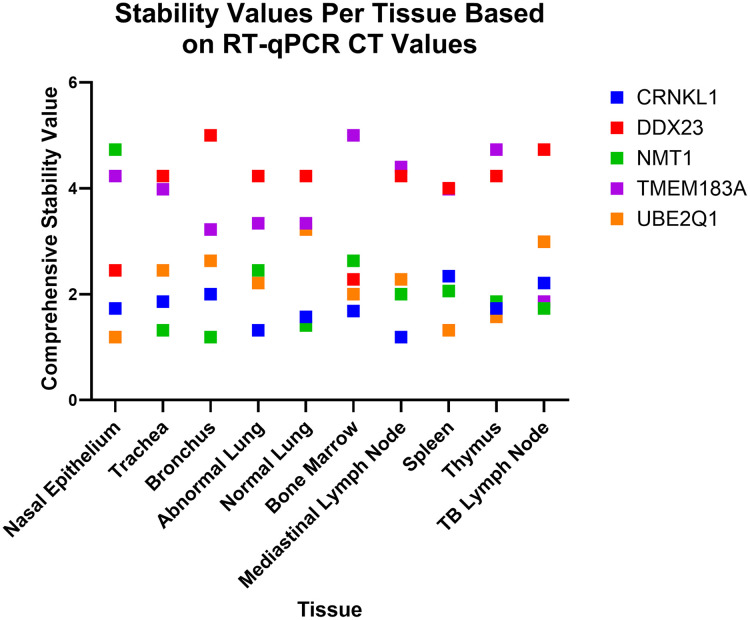
Stability values of candidate reference genes in each tissue tested. Fig 6 demonstrates the comprehensive stability values calculated by RefFinder for the 5 candidate reference genes in each tissue tested. See Table 3 for numerical values of the comprehensive stability values.

To assess the suitability of using publicly available transcriptomes for identifying reference genes, the stability of the proposed reference gene candidates was explored in bovine respiratory and lymphoid tissues from both healthy and BRSV-infected calves. Since only two healthy animals were included, the results must be interpreted conservatively. Samples included lymphoid tissues, including bone marrow, tracheobronchial and mediastinal lymph nodes, spleen, and thymus, and respiratory tissues, including nasal epithelium, trachea, bronchus, and cranioventral and caudodorsal lung sections. RT-qPCR results for respiratory tissues and lymphoid tissues are presented in Fig 5A and 5B, respectively, with seven infected calves indicated by circles and two healthy calves indicated by squares. TMEM183A was the least abundant gene across all tissue types. Using RefFinder to analyze raw C_t_ values for each tissue in both healthy and BRSV-infected calves, the stability ranking of these genes for each tissue was calculated and shown in Fig 6 and Table 3. CRNKL1 ranked among the top two most stable genes in most tissues, except spleen and tracheobronchial lymph node. While CRNKL1 was the most stable in many tissues according to MIQE guidelines, it is recommended to use 2–3 reference genes for normalization [37]. Due to fluctuations in each gene’s ranking in different tissues, stability values are reported individually by tissue.

**Table 3:** Stability values per tissue based on RT-qPCR CT values.

Tissue	CRNKL1	DDX23	NMT1	TMEM183A	UBE2Q1
Nasal Epithelium	1.73	2.45	4.73	4.23	1.19
Trachea	1.86	4.23	1.32	3.98	2.45
Bronchus	2	5	1.19	3.22	2.63
Abnormal Lung	1.32	4.23	2.45	3.34	2.21
Normal Lung	1.57	4.23	1.41	3.34	3.22
Bone Marrow	1.68	2.28	2.63	5	2
Mediastinal Lymph Node	1.19	4.23	2	4.4	2.28
Spleen	2.34	4	2.06	3.98	1.32
Thymus	1.73	4.23	1.86	4.73	1.57
TB Lymph Node*	2.21	4.73	1.73	1.86	2.99

- These RefFinder comprehensive stability values are computed by calculating the geometric mean of ranks of the stability values from BestKeeper, DeltaCT, GeNorm, and NormFinder.

*Tracheobronchial lymph node

## Discussion

RT-qPCR is an essential technique for studying gene regulation. Despite numerous studies and the MIQE guidelines highlighting the proper use of reference genes, the use of a single unvalidated housekeeping gene remains a common practice in published literature [37]. However, the validation and use of multiple reference genes are gaining momentum, with not only more published data but also the establishment of databases such as “Internal Control Genes for RT-qPCR Normalization” since 2018 [38]. Although many studies identify bovine reference genes, the majority focus on cells within the blood, gastrointestinal, or reproductive tracts [9,10,39]. Consequently, to our knowledge, no validation studies of reference genes have been published for respiratory or lymphoid tissues in cattle. Thus, this study aims to achieve two goals: first, to use a bioinformatics pipeline leveraging publicly available transcriptomes to identify candidate reference genes for RT-qPCR; second, to explore the stability of these reference genes across multiple tissues from healthy calves and calves experimentally infected with a common respiratory virus, BRSV. By using publicly accessible transcriptomic data, the study provides an easy method to identify candidate reference genes. To our knowledge, this is the first report using publicly available transcriptomes to discover and design novel reference genes in *Bos taurus* tissues.

The first goal of this study was to identify novel candidate reference genes from publicly accessible transcriptomes, using RefFinder to determine the most stably expressed genes across a tissue panel (Table 1). It was of interest to utilize healthy tissue transcriptomes to enhance the applicability of bovine RT-qPCR analyses. A limitation of using publicly available transcriptomes was that, at the time, only healthy tissues were available, and not only were we limited to 16 animals, but also some tissues were represented only once. However, because most transcriptomes overlapped in the PCA plots, it was decided to use all available transcriptomes. Of the 22 candidate reference genes identified, only two have been previously used as reference genes, and their stability has been published. In previous work, our lab employed a similar bioinformatic method to validate RNA-seq data, and it was also found that UBE2Q1 was stable across the transcriptomes of blood from both healthy and diseased cattle [21]. Also, ARIH2 has been published for use with human gene expression analyses [40].

While the 5 candidate reference genes described in this report were stable in the tissues from the BRSV-infected and healthy calves tested, several of these genes have been previously identified as differentially expressed within certain experimental conditions in non-bovine species. However, only one of these genes was shown to be upregulated in disease in cattle: NMT1, which was described to be upregulated in lung tissue during Mannheimiosis [41]. In other research, SPOP, NMT1, PIGU, YKT6, MTERF3, CERS5, RANBP9, DDX23, UBE2Q1, GLN2, and FBXW11 were demonstrated in human cancer studies to be upregulated, downregulated, or mutated [42–51]. This apparent discrepancy between our findings and previous reports indicating that some of the candidate reference genes reported here were not stably expressed in certain disease states reinforces the fact that there is no single reference gene that is appropriate for use in all research. For example, while NMT1 was demonstrated here to be stably expressed in BRSV-infected and healthy calves, the previous report of its upregulation during Mannheimiosis indicates that it would not be a suitable reference gene in studies of that disease in cattle.

It was noteworthy that the commonly utilized reference genes GAPHD and ACTB were not found among the top 2000 least variably expressed genes in the data set, based on coefficient of variation among published transcriptomes. This aligns with a few studies that have expressed concerns about their lack of stability, yet continued to use them as reference genes [6,17]. Ultimately, this bioinformatic analysis yielded a set of novel candidate reference genes, although it will be important for other researchers who wish to use these reference genes to confirm their stability in the tissue or disease state they are studying. Our development and use of primer sets for the identified stable reference genes will facilitate their use in future work by researchers conducting cattle health research using relative quantification by RT-qPCR.

To explore the stability of these five candidate bovine reference genes, they were evaluated in a convenient set of samples collected from calves in an unrelated study conducted to characterize the response of calves to BRSV challenge. However, in that study, negative control animals were not included. Therefore, in order to evaluate the stability of the candidate reference genes in BRSV-challenged cattle relative to non-infected cattle, two age-matched healthy calves were purchased and euthanized to provide tissues for comparison. This is a limitation of this study, because the age-matched healthy calves were managed differently than the BRSV-challenged calves; they were sampled at a different time, and only 2 healthy calves were included. While the stable expression of the 5 candidate genes demonstrated in Figs 5 and 6 supports the concept that these are appropriate reference genes for studies of differential gene expression in cattle, at least during BRSV infection, the results must be considered with this study limitation in mind. The candidate reference genes presented here merit consideration for use in future research of differential gene expression in cattle, but investigators will need to confirm the stability of expression of these genes in their own model [37].

This study demonstrates a relatively easy-to-use method using publicly available transcriptomic data as a starting point for identifying more appropriate reference genes for gene expression analysis. Five reference gene candidates are presented with specific primers and adequate efficiencies in one example experimental condition. While working primer sets to quantify mRNA expression for five proposed reference genes are presented here, it will be important for researchers wishing to use these reference genes to confirm that their expression is stable in the system they study [37]. These are not universal reference genes, as, to date, no gene that is stable in all tissues in all disease states has been identified for any species, but they are a starting point for future bovine gene expression analyses.

## Supporting information

S1 Table Gene functions. This table describes the gene functions of the top 22 most stably expressed genes.
(XLSX)

S2 Table Transcriptomic data sets. Counts per million of all genes within each transcriptomic data set.
(XLSX)

S3 Table Stability values for top 200 most stable genes. Stability values determined by each program for the top 200 most stable genes, and the comprehensive stability values for the top 200 most stable genes.
(XLSX)

S4 Table RNA quantity and quality assessment. Results of quantity and quality assessment of RNA extracted from tissues of healthy and BRSV-infected calves. If N/A is present, there was insufficient volume to obtain nanodrop-quality values.
(XLSX)

S1 File Description of reasons for failed primer designs. Files describing the problems encountered when developing primer sets for certain candidate reference genes.
(DOCX)

S1 Fig Melting plots per gene. Each of the following graphs show the melting curves of triplicate wells of each gene: A CRNKL1, B DDX23, C NMT1, D TMEM183A, and E UBE2Q1.
(TIF)

## References

[1] The correlation between expression profiles measured in single cells and in traditional bulk samples | Scientific Reports. [cited 8 Feb 2026]. https://www.nature.com/articles/srep37022 10.1038/srep37022 PMC5111061 27848982

[2] Livak KJ, Schmittgen TD. Analysis of relative gene expression data using real-time quantitative PCR and the 2(-Delta Delta C(T)) Method. Methods. 2001;25(4):402–8. doi: 10.1006/meth.2001.1262 11846609

[3] Pfaffl MW. A new mathematical model for relative quantification in real-time RT–PCR. Nucleic Acids Res. 2001. 10.1093/nar/29.9.e45 PMC55695 11328886

[4] Joshi CJ, Ke W, Drangowska-Way A, O’Rourke EJ, Lewis NE. What are housekeeping genes?. PLOS Computational Biology. 2022;18:e1010295. doi: 10.1371/journal.pcbi.1010295 PMC9312424 35830477

[5] Kadegowda AKG, Bionaz M, Thering B, Piperova LS, Erdman RA, Loor JJ. Identification of internal control genes for quantitative polymerase chain reaction in mammary tissue of lactating cows receiving lipid supplements. J Dairy Sci. 2009;92(5):2007–19. doi: 10.3168/jds.2008-1655 19389958

[6] Warrington JA, Nair A, Mahadevappa M, Tsyganskaya M. Comparison of human adult and fetal expression and identification of 535 housekeeping/maintenance genes. Physiol Genomics. 2000;2(3):143–7. doi: 10.1152/physiolgenomics.2000.2.3.143 11015593

[7] Coelho TC, Chalfun-Junior A, Barreto HG, Duarte M de S, Garcia B de O, Teixeira PD. Reference gene selection for quantitative PCR in liver, skeletal muscle, and jejunum of Bos indicus cattle. R Bras Zootec. 2022;51. doi: 10.37496/rbz5120210120

[8] Bonnet M, Bernard L, Bes S, Leroux C. Selection of reference genes for quantitative real-time PCR normalisation in adipose tissue, muscle, liver and mammary gland from ruminants. Animal. 2013;7(8):1344–53. doi: 10.1017/S1751731113000475 23552195

[9] Spalenza V, Girolami F, Bevilacqua C, Riondato F, Rasero R, Nebbia C, et al. Identification of internal control genes for quantitative expression analysis by real-time PCR in bovine peripheral lymphocytes. Vet J. 2011;189(3):278–83. doi: 10.1016/j.tvjl.2010.11.017 21169039

[10] Pfaffl MW, Tichopad A, Prgomet C, Neuvians TP. Determination of stable housekeeping genes, differentially regulated target genes and sample integrity: BestKeeper--Excel-based tool using pair-wise correlations. Biotechnol Lett. 2004;26(6):509–15. doi: 10.1023/b:bile.0000019559.84305.47 15127793

[11] Zhou Z, Cong P, Tian Y, Zhu Y. Using RNA-seq data to select reference genes for normalizing gene expression in apple roots. PLoS One. 2017;12(9):e0185288. doi: 10.1371/journal.pone.0185288 28934340 PMC5608369

[12] Yang H, Liu J, Huang S, Guo T, Deng L, Hua W. Selection and evaluation of novel reference genes for quantitative reverse transcription PCR (qRT-PCR) based on genome and transcriptome data in Brassica napus L. Gene. 2014;538(1):113–22. doi: 10.1016/j.gene.2013.12.057 24406618

[13] Jureckova K, Raschmanova H, Kolek J, Vasylkivska M, Branska B, Patakova P, et al. Identification and Validation of Reference Genes in Clostridium beijerinckii NRRL B-598 for RT-qPCR Using RNA-Seq Data. Front Microbiol. 2021;12:640054. doi: 10.3389/fmicb.2021.640054 33815328 PMC8012504

[14] Hoang VLT, Tom LN, Quek X-C, Tan J-M, Payne EJ, Lin LL, et al. RNA-seq reveals more consistent reference genes for gene expression studies in human non-melanoma skin cancers. PeerJ. 2017;5:e3631. doi: 10.7717/peerj.3631 28852586 PMC5572537

[15] Nagalakshmi U, Wang Z, Waern K, Shou C, Raha D, Gerstein M, et al. The transcriptional landscape of the yeast genome defined by RNA sequencing. Science. 2008;320(5881):1344–9. doi: 10.1126/science.1158441 18451266 PMC2951732

[16] Andersen CL, Jensen JL, Ørntoft TF. Normalization of real-time quantitative reverse transcription-PCR data: A model-based variance estimation approach to identify genes suited for normalization, applied to bladder and colon cancer data sets. Cancer Res. 2004;64(15):5245–50. doi: 10.1158/0008-5472.CAN-04-0496 15289330

[17] Vandesompele J, De Preter K, Pattyn F, Poppe B, Van Roy N, De Paepe A, et al. Accurate normalization of real-time quantitative RT-PCR data by geometric averaging of multiple internal control genes. Genome Biol. 2002;3(7):RESEARCH0034. doi: 10.1186/gb-2002-3-7-research0034 12184808 PMC126239

[18] Silver N, Best S, Jiang J, Thein SL. Selection of housekeeping genes for gene expression studies in human reticulocytes using real-time PCR. BMC Mol Biol. 2006;7:33. doi: 10.1186/1471-2199-7-33 17026756 PMC1609175

[19] Xie F, Xiao P, Chen D, Xu L, Zhang B. miRDeepFinder: A miRNA analysis tool for deep sequencing of plant small RNAs. Plant Mol Biol. 2012. doi: 10.1007/s11103-012-9885-2 22290409

[20] NCBI GEO: archive for functional genomics data sets—update | Nucleic Acids Research | Oxford Academic. [cited 5 Mar 2025]. https://academic.oup.com/nar/article/41/D1/D991/1067995?login=true 10.1093/nar/gks1193 PMC3531084 23193258

[21] Koufariotis LT, Chen Y-PP, Chamberlain A, Vander Jagt C, Hayes BJ. A catalogue of novel bovine long noncoding RNA across 18 tissues. PLoS One. 2015;10(10):e0141225. doi: 10.1371/journal.pone.0141225 26496443 PMC4619662

[22] Tizioto PC, Kim J, Seabury CM, Schnabel RD, Gershwin LJ, Van Eenennaam AL, et al. Immunological response to single pathogen challenge with agents of the bovine respiratory disease complex: An RNA-sequence analysis of the bronchial lymph node transcriptome. PLoS One. 2015;10(6):e0131459. doi: 10.1371/journal.pone.0131459 26121276 PMC4484807

[23] Rosen BD, Bickhart DM, Schnabel RD, Koren S, Elsik CG, Tseng E. De novo assembly of the cattle reference genome with single-molecule sequencing. Gigascience. 2020. 10.1093/gigascience/giaa021 PMC7081964 32191811

[24] Fang L, Cai W, Liu S, Canela-Xandri O, Gao Y, Jiang J, et al. Comprehensive analyses of 723 transcriptomes enhance genetic and biological interpretations for complex traits in cattle. Genome Res. 2020;30(5):790–801. doi: 10.1101/gr.250704.119 32424068 PMC7263193

[25] Harhay GP, Smith TP, Alexander LJ, Haudenschild CD, Keele JW, Matukumalli LK, et al. An atlas of bovine gene expression reveals novel distinctive tissue characteristics and evidence for improving genome annotation. Genome Biol. 2010;11(10):R102. doi: 10.1186/gb-2010-11-10-r102 20961407 PMC3218658

[26] Johnston D, Earley B, McCabe MS, Lemon K, Duffy C, McMenamy M, et al. Experimental challenge with bovine respiratory syncytial virus in dairy calves: Bronchial lymph node transcriptome response. Sci Rep. 2019;9(1):14736. doi: 10.1038/s41598-019-51094-z 31611566 PMC6791843

[27] Andrews. FastQC: A Quality Control Tool for High Throughput Sequence Data. https://www.bioinformatics.babraham.ac.uk/projects/fastqc/. Accessed 2025 March 4.

[28] Bolger AM, Lohse M, Usadel B. Trimmomatic: A flexible trimmer for Illumina sequence data. Bioinformatics. 2014;30(15):2114–20. doi: 10.1093/bioinformatics/btu170 24695404 PMC4103590

[29] Dobin A, Davis CA, Schlesinger F, Drenkow J, Zaleski C, Jha S, et al. STAR: Ultrafast universal RNA-seq aligner. Bioinformatics. 2013;29(1):15–21. doi: 10.1093/bioinformatics/bts635 23104886 PMC3530905

[30] Dobin A, Gingeras TR. Mapping RNA-seq Reads with STAR. Curr Protoc Bioinformatics. 2015;51:11.14.1-11.14.19. doi: 10.1002/0471250953.bi1114s51 26334920 PMC4631051

[31] R Core Team. R: A language and environment for statistical computing. https://www.r-project.org/. 2021. Accessed 2025 March 4.

[32] Zhang Y, Parmigiani G, Johnson WE. ComBat-seq: Batch effect adjustment for RNA-seq count data. NAR Genom Bioinform. 2020;2(3):lqaa078. doi: 10.1093/nargab/lqaa078 33015620 PMC7518324

[33] Chen Y, Lun ATL, Smyth GK. From reads to genes to pathways: Differential expression analysis of RNA-Seq experiments using Rsubread and the edgeR quasi-likelihood pipeline. F1000Research. 2016. doi: 10.12688/f1000research.8987.2 PMC4934518 27508061

[34] Anders S, Huber W. Differential expression analysis for sequence count data. Genome Biol. 2010;11(10):R106. doi: 10.1186/gb-2010-11-10-r106 20979621 PMC3218662

[35] Scott MA, Woolums AR, Swiderski CE, Perkins AD, Nanduri B, Smith DR, et al. Whole blood transcriptomic analysis of beef cattle at arrival identifies potential predictive molecules and mechanisms that indicate animals that naturally resist bovine respiratory disease. PLoS One. 2020;15(1):e0227507. doi: 10.1371/journal.pone.0227507 31929561 PMC6957175

[36] Ye J, Coulouris G, Zaretskaya I, Cutcutache I, Rozen S, Madden TL. Primer-BLAST: A tool to design target-specific primers for polymerase chain reaction. BMC Bioinformatics. 2012;13:134. doi: 10.1186/1471-2105-13-134 22708584 PMC3412702

[37] Bustin SA, Benes V, Garson JA, Hellemans J, Huggett J, Kubista M, et al. The MIQE guidelines: Minimum information for publication of quantitative real-time PCR experiments. Clin Chem. 2009;55(4):611–22. doi: 10.1373/clinchem.2008.112797 19246619

[38] Sang J, Wang Z, Li M, Cao J, Niu G, Xia L, et al. ICG: A wiki-driven knowledgebase of internal control genes for RT-qPCR normalization. 2018. 10.1093/nar/gkx875 PMC5753184 29036693

[39] Die JV, Baldwin RL, Rowland LJ, Li R, Oh S, Li C, et al. Selection of internal reference genes for normalization of reverse transcription quantitative polymerase chain reaction (RT-qPCR) analysis in the rumen epithelium. PLoS One. 2017;12(2):e0172674. doi: 10.1371/journal.pone.0172674 28234977 PMC5325532

[40] Tirado-Hurtado I, Araujo JM, Saravia CH, Requena M, Rolfo CD, Raez LE. Selection of highly stable genes for the transcriptomic evaluation of lung cancer. J Clin Oncol. 2018. doi: 10.1200/JCO.2018.36.15_suppl.e24318

[41] Shrivastav A, Suri SS, Mohr R, Janardhan KS, Sharma RK, Singh B. Expression and activity of N-myristoyltransferase in lung inflammation of cattle and its role in neutrophil apoptosis. Vet Res. 2010;41(1):9. doi: 10.1051/vetres/2009057 19796608 PMC2775168

[42] Ding D, Song T, Jun W, Tan Z, Fang J. Decreased expression of the SPOP gene is associated with poor prognosis in glioma. Int J Oncol. 2015;46(1):333–41. doi: 10.3892/ijo.2014.2729 25351530

[43] Sun Y, Guan Z, Sheng Q, Duan W, Zhao H, Zhou J, et al. N-myristoyltransferase-1 deficiency blocks myristoylation of LAMTOR1 and inhibits bladder cancer progression. Cancer Lett. 2022;529:126–38. doi: 10.1016/j.canlet.2022.01.001 34999170

[44] Wu J, Zhang T, Xiong H, Zeng L, Wang Z, Peng Y, et al. Tumor-Infiltrating CD4+ central memory T cells correlated with favorable prognosis in oral squamous cell carcinoma. J Inflamm Res. 2022;15:141–52. doi: 10.2147/JIR.S343432 35035226 PMC8754505

[45] Wang W-S, Zi J-J, Sun M-T, Mei W, Li S-F, Yang N, et al. Expression of MTERF3 gene in breast carcinoma and the relationship with clinicopathological characteristics. Transl Cancer Res. 2020;9(1):173–86. doi: 10.21037/tcr.2019.12.65 35117171 PMC8799005

[46] Mojakgomo R, Mbita Z, Dlamini Z. Linking the ceramide synthases (CerSs) 4 and 5 with apoptosis, endometrial and colon cancers. Exp Mol Pathol. 2015;98(3):585–92. doi: 10.1016/j.yexmp.2015.03.019 25779024

[47] Wu G, Li J, Qin C. Reduced RANBP9 expression is associated with poor prognosis in colorectal cancer patients. Transl Cancer Res. 2019;8(8):2704–12. doi: 10.21037/tcr.2019.10.24 35117028 PMC8797687

[48] Zhang B, Deng C, Wang L, Zhou F, Zhang S, Kang W. Upregulation of UBE2Q1 via gene copy number gain in hepatocellular carcinoma promotes cancer progression through β‐catenin‐EGFR‐PI3K‐Akt‐mTOR signaling pathway. 2025. doi: 10.1002/mc.22747 29027712

[49] Dong Y, Cai Q, Fu L, Liu H, Ma M, Wu X. Study of the G protein nucleolar 2 value in liver hepatocellular carcinoma treatment and prognosis. Biomed Res Int. 2021;2021:4873678. doi: 10.1155/2021/4873678 34337013 PMC8315868

[50] Zhao C, Li Y, Qiu C, Chen J, Wu H, Wang Q, et al. Splicing factor DDX23, transcriptionally activated by E2F1, promotes ovarian cancer progression by regulating FOXM1. Front Oncol. 2021;11:749144. doi: 10.3389/fonc.2021.749144 34966670 PMC8710544

[51] Chen C, Zhou H, Zhang X, Liu Z, Ma X. Association of FBXW11 levels with tumor development and prognosis in chondrosarcoma. Cancer Biomark. 2022;35(4):429–37. doi: 10.3233/CBM-210426 36404534 PMC12364250

