# Supplementary material for: Identification and validation of novel reference genes for bovine respiratory and lymphoid tissues using public transcriptomes and BRSV challenge model
Source: PLoS One. 2026 Jul 21;21(7):e0352137. doi: 10.1371/journal.pone.0352137 (PMC13387523; doi:10.1371/journal.pone.0352137)
Supplement: S1 File — Files describing the problems encountered when developing primer sets for certain candidate reference genes. (DOCX) [file pone.0352137.s005.docx]

**Barber et al.,** "**Identification and Validation of Novel Reference Genes for Bovine Respiratory and Lymphoid Tissues Using Public Transcriptomes and BRSV Challenge Model"**

**Problems encountered when developing primer sets for certain candidate reference genes**

Two primers sets tested for SPOP demonstrated nonspecific binding, yielding multiple bands on gel electrophoresis after classical RT-PCR. For YKT6, both primer sets exhibited nonspecific binding, producing bands larger than 1000bp and amplifying in the no reverse transcriptase control during RT-qPCR. Primer sets tested for MTERF4 also bound in the no reverse transcriptase control, with a visible amplicon on gel electrophoresis. For CERS5, the first primer set did not produce an amplicon in classical RT-PCR, while the second and third primer sets showed primer dimer formation, indicated by bright bands on gel and two peaks on melt curve during RT-qPCR. Adjusting the concentrations of the forward and reverse primers for CERS5 primer two did not improve results, as two peaks persisted on the melt curve. The TTC4 primer set was excluded because, in neutrophil samples, the raw CTs were over 38 using 10 ng of cDNA per well. Lastly, one of the GLN2 primer sets tested produced multiple amplicons in classical RT-PCR, while the second set tested bound to the no reverse transcriptase control.
