# Supplementary figures and images for: Identification and validation of novel reference genes for bovine respiratory and lymphoid tissues using public transcriptomes and BRSV challenge model
Source: PLoS One. 2026 Jul 21;21(7):e0352137. doi: 10.1371/journal.pone.0352137 (PMC13387523; doi:10.1371/journal.pone.0352137)

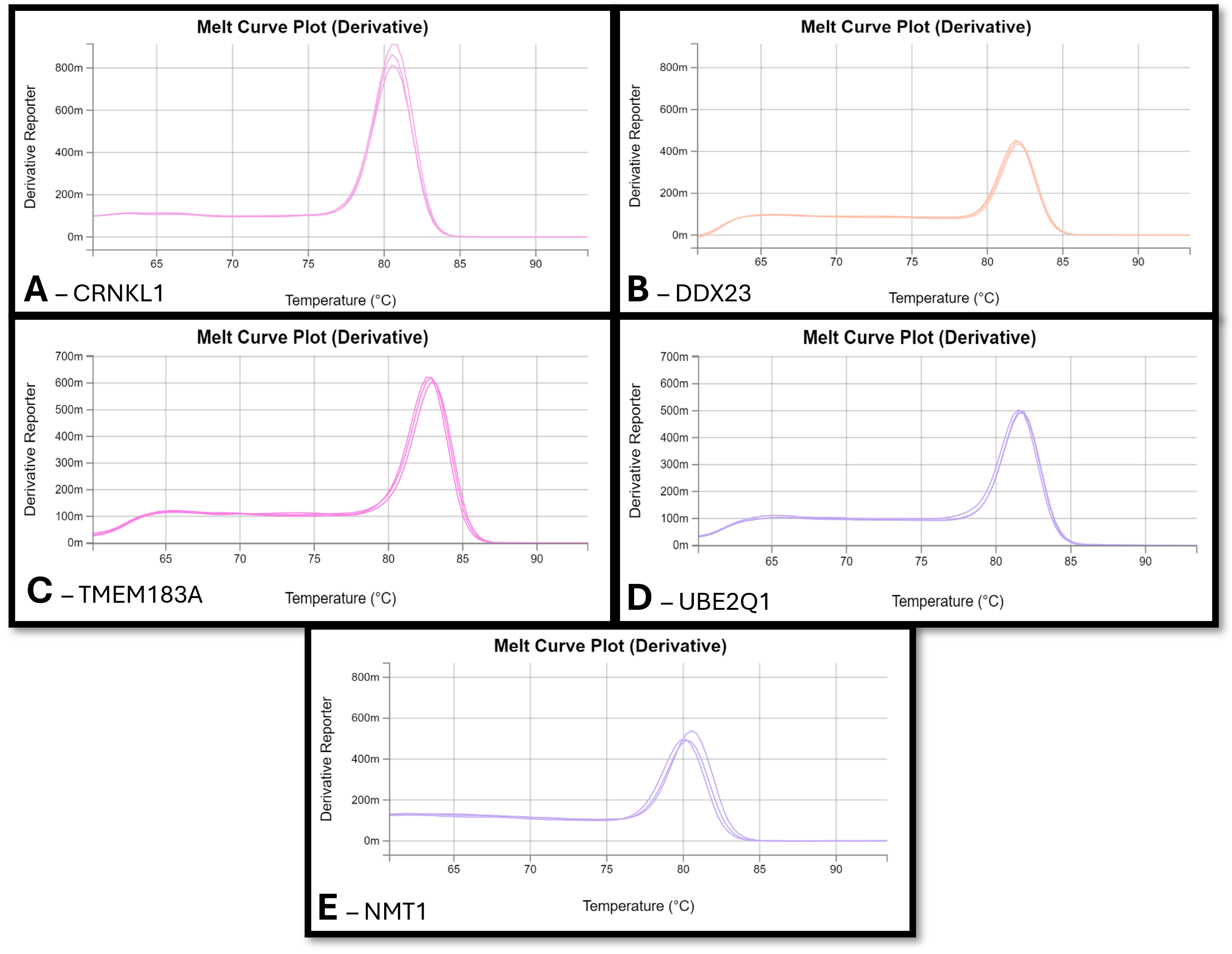

Supplement: S1 Fig — Each of the following graphs show the melting curves of triplicate wells of each gene: A CRNKL1, B DDX23, C NMT1, D TMEM183A, and E UBE2Q1. (TIF) [file pone.0352137.s006.tif]
